# Serum Endostatin Levels in Oral Squamous Cell Carcinoma

**Published:** 2018-05

**Authors:** Maryam Mardani, Azadeh Andishe Tadbir, Mohammad Ali Ranjbar, Bijan Khademi, Mohammad Javad Fattahi, Ahmad Rahbar

**Affiliations:** 1 *Oral and Dental Disease Research Center* *, School of Dentistry* *, Shiraz University of Medical Sciences, Shiraz, Iran.*; 2 *Department of Oral and Maxillofacial Pathology, School of Dentistry, Shiraz University of Medical Sciences, Shiraz, Iran.*; 3 *Department of Otolaryngology, Khalili Hospital, Shiraz University of Medical Sciences, Shiraz, Iran.*; 4 *Shiraz Institute for Cancer Research, Shiraz University of Medical Sciences, Shiraz, Iran.*; 5 *Undergraduate Student, School of Dentistry, Shiraz University of Medical Sciences, Shiraz, Iran.*

**Keywords:** Endostatin, Mouth, Serum, Squamous Cell Carcinoma.

## Abstract

**Introduction:**

Endostatin is a C-terminal proteolytic fragment of collagen XVIII and, as with angiostatin and thrombospondin, is known as an antiangiogenic agent. The aim of this study was to assess the level of serum endostatin in patients with oral squamous cell carcinoma (SCC), and its association with the clinicopathological characteristics of the tumor.

**Materials and Methods:**

Using an enzyme-linked immunosorbent assay (ELISA) kit, we investigated the circulating levels of endostatin in the blood serum of 45 patients with oral SCC and 45 healthy controls.

**Results:**

The mean level of serum endostatin in patients was significantly lower (68.8±85 ng/ml) than in healthy controls (175.6±73 ng/ml) (P<0.001). Serum endostatin levels were significantly lower in patients with lymph node metastasis compared with patients without lymph node metastasis (P<0.001). In addition, serum endostatin level was associated with higher histological grade (P<0.001). There were no apparent correlations between serum endostatin concentration and clinicopathological features such as age, gender, and tumor stage (P>0.05).

**Conclusion:**

Findings of the present study suggest the prognostic and anti-metastatic role of endostatin, and this may be used as a tool for monitoring tumor progression.

## Introduction

Oral squamous cell carcinoma (SCC) is the sixth most common malignancy reported in the world, and has the highest mortality rate due to malignancies (1). Research is being undertaken into the early diagnosis and prevention of this deadly cancer in order to improve outcomes.

Although histopathologic evaluation is the gold standard for the diagnosis of oral cancers, other pathological techniques such as an assessment of abnormally increased levels of proteins may be useful for optimal diagnosis and treatment. These markers are currently being used to predict the biological behavior and prognosis of oral malignancy.

Interest in endostatin, an anti-tumor molecule in cancers, has increased over the last two decades. Endostatin is described as a 22 kDa C-terminal fragment of collagen XVIII, which is a vascular and epithelial basement membrane protein(2,3).Previous studies have demonstrated various functions of this protein. For example, endostatin can cause apoptosis of the endothelial cells and serves as a suppressor of endothelial cell migration and tumor growth (4–6). Indeed, endostatin interferes with tumor progression by preventing the activity of tumor growth-stimulating factors (7,8). Furthermore, a few studies have reported that endostatin inhibits tumor metastasis by limiting blood supply, and therefore is considered a potential anti-tumor marker in malignant tumor treatment (9,10). To date, many studies have investigated endostatin levels in different types of cancers such as soft tissue sarcoma, renal cell carcinoma, and ovarian carcinoma (11-13). In addition, the association of endostatin level with prognosis and aggressiveness of tumors has been confirmed. It has already been established that patients with poor prognosis cancers have higher serum levels of endostatin (14-16). Investigations into the prognostic significance of circulating endostatin levels have demonstrated the association of higher serum levels of endostatin with progressive and poorly differentiated colorectal and bladder cancers (17,18). It is therefore reasonable to assume that serum endostatin levels can be used as a biomarker for diagnosing and predicting the prognosis of cancers. This study was conducted to investigate the potential role of serum endostatin levels in head and neck SCC as well as the correlation between serum endostatin levels and various clinical and pathological features.

## Materials and Methods

The medical records of 45 patients (22 men and 23 women; mean age, 57±16 years) with a histologically proven diagnosis of oral SCC were reviewed. Blood samples were obtained from the archives of the laboratory of pathology, Khalili Hospital of Shiraz University of Medical Sciences. Cases with other tumors, inflammation or infections were excluded from the study. In the control group, 45 normal subjects (22 men and 23 women; mean age, 56.6±15 years) with no evidence of inflammatory or systemic diseases were enrolled. All volunteers were fully informed about the study protocol and objectives, and signed an informed consent form to participate in the study. Two pathologists confirmed the diagnosis of oral SCC. Clinical staging of cases was determined according to the tumor, node, metastasis (TNM) staging system of the American Joint Committee on Cancer (AJCC) (19). The histopathological grade of oral SCC was determined based on World Health Organization (WHO) criteria.

Collection of blood samples was performed with commercially available ethylenediamin- etetraacetic acid (EDTA) blood collection tubes (Greiner Bio-One GmbH, Kremsmunster, Austria) before surgery. 

Serum was separated from clotted blood after centrifugation at 4°C and stored at −70°C for future assay. Endostatin concentrations were determined by enzyme-linked immunosorbent assay (ELISA) according to the manufacturer's instructions (R&D Systems Inc, Minneapolis, MNSS 413, USA).When primary radical surgery was performed, the patients were treated with combined primary radiotherapy and chemotherapy.

*Statistical analysis*

All statistical analyses were performed using SPSS 19.0 software. Comparisons between patients and controls and comparisons among patients were performed using parametric (Student’s t) and nonparametric (Mann-Whitney and Kruskal-Wallis) tests,respectively. Correlation between variables were determined and p-values less than 0.05 were considered statistically significant.

## Results

Clinicopathological and demographic data are summarized in Table 1. All patients had localized tumor (M0). The mean serum endostatin level in patients with SCC (68.8±85 ng/ml) was significantly lower than that in healthy controls (175.6±73 ng/ml) (P<0.001). A positive correlation was seen between the serum levels of endostatin and the histological grade of tumors (P<0.001). The serum levels of endostatin were significantly lower in patients with lymph node metastasis (N^0^ group) compared with those without metastasis (N^+^ group) (P<0.001). There was no significant correlation between serum endostatin level and other clinicopathological variables such as age, gender, and tumor stage (P>0.05) (Table.1).

**Table 1:** Clinicopathological profile of 45 oral SCC patients and correlation with endostatin serum levels

	**Feature **	**No. (%)**	**Mean serum level (Mean±SD)**	**P-value**
Gender	Male Female	20 (45.5) 25 (55.5)	55.38±14.77 58.19±15.61	0.67
Regional lymph node involvement	N^0^ N^+^	17 (37.8) 28 (62.2)	72.65±8.92 45.46±4.63	0.000
Age	<65 ≥65	20 (45.5) 25 (55.5)	55.48±15.28 58.11±15.23	0.92
Tumor stage	I, II III, IV	14 (31.1) 31 (68.8)	53.54±13.65 61.8± 15.98	0.07
Histological grade	Grade I, II Grade III, IV	22 (49.9) 23 (51.1)	43.32±2.91 78.19±4.87	0.000

## Discussion

Several studies have demonstrated that angiogenesis and its inhibitors have a crucial role in the pathogenesis of malignancies and can affect tumor progression, invasion, and eventually lymph node metastasis (20, 21). Furthermore, several angiogenesis-related factors such as endostatin, have been considered as tumor prognostic markers. Therefore, in this present study we evaluated the relationship between serum endostatin levels and clinicopathological characteristics in oral SCC.

We found statistically significant differences in endostatin levels between patients and controls (P<0.001). This finding is consistent with a previous study which investigated the prognostic value of plasma endostatin levels in head and neck SCC, and reported significantly lower endostatin levels in patients with cancer versus healthy volunteers (22). In our study, the serum levels of endostatin were lower in patients with nodal metastasis (N^+^group) compared with patients without nodal metastasis (N^0^group). 

This finding may represent the anti-metastatic role of this marker. It is suggested that the decrement of serum endostatin levels in patients with lymph node metastasis is related to lower production of the precursor of endostatin, collagen XVIII (23).

Our result is also in agreement with a similar study which investigated the association of endostatin on tumor growth and lymph node metastasis in animals implanted with oral cancer cells, and revealed that endostatin may inhibit growth and lymph node metastasis in these cancers (24). 

Similarly, another investigation which analyzed the immunohistochemical expression of endostatin in oral SCC showed a lower expression of endostatin in the tumors of cases with multiple metastatic lymph nodes compared with non-metastatic tumors. Therefore, a reverse correlation between the tissue expression of endostatin and lymph node metastasis was suggested (23).

According to the available evidence, the molecular mechanisms by which endostatin exerts its inhibitory effect has not yet been fully elucidated. VEGF-C expression in a variety of human cancers and the correlation of this protein with the rate of lymph node metastasis have already been demonstrated (25–28). Hence, higher endostatin levels in patients without lymph node metastasis may be due to the endostatin effect on lymph expansion, which can inhibit lymph angiogenesis by down-regulating VEGF-C expression in tumor cells (24). In this regard, Shao et al. reported that recombinant endostatin inhibited the proliferation and migration of lymphatic endothelial cells *in vitro *(29). Taking these observations together, it can be proposed that elevated endostatin levels can suppress tumor lymph angiogenesis, which subsequently reduces lymph node metastasis.

In contrast with our findings, however, Feldman et al. showed increased levels of plasma endostatin in colorectal patients with liver metastasis (11). It has been demonstrated that although endostatin is a potent antiangiogenic substance in tumor lymph node involvement, circulating endostatin levels might not be sufficient to push the angiogenic balance toward antiangiogenesis (30).

Previous investigations have revealed that the effect of endostatin on endothelial cells depends on the length of exposure (31). Moreover, the concentration of endostatin is important in terms of the optimal inhibitory effect. Celik et al. demonstrated that higher and lower dosages of endostatin had less inhibitory action on lymph node involvement (32). Another reason for conflicting findings between studies in different cancers may be the differences in angiogenic pathways in distinct types of tumor (33).

It has already been shown that endostatin inhibits the activation of matrix metalloprotease 2 and 9, which are closely associated with metastatic potential and invasion of tumoral cells (34-36). Therefore, increases in the amount of endostatin may suppress angiogenesis, lymph angiogenesis, and progression of tumor by inhibiting the matrix metalloprotease 2 and 9.

Another finding of the present study is the association of increased endostatin levels with higher histological grades of oral SCC. Our result is consistent with a study that demonstrated a significant association between higher plasma levels of endostatin and poorer tumor grade of oral SCC (22). The association of higher serum endostatin levels and poorer patient prognosis in non-small cell lung cancer, soft tissue sarcoma, and bladder cancer has also been demonstrated (11,15,18).

Combining antiangiogenic factors such as endostatin with chemotherapy is one of the most effective ways to increase the survival rate of patients with cancer (37). Therefore, the therapeutic properties of endostatin as an antiangiogenic drug must be further investigated. In addition, future randomized prospective studies should be conducted with a larger sample size so that a significant association between serum endostatin levels and other clinicopathological variables may be confirmed.

## Conclusion

Based on the findings of this study, the serum levels of endostatin were significantly lower in patients with lymph node metastasis. In addition, there was a positive correlation between endostatin level and tumor grade. Therefore, serum levels of endostatin may help clinicians predict the biological behavior of oral SCC.
